# A novel frameshift variant in the *MED13* gene causing intellectual developmental disorder-61 in a Chinese family

**DOI:** 10.3389/fped.2025.1699544

**Published:** 2025-10-21

**Authors:** Qi Yang, Qiang Zhang, Sheng Yi, Gaojie Huang, Xunzhao Zhou, Shang Yi, Jingsi Luo

**Affiliations:** ^1^ Guangxi Key Laboratory of Birth Defects Research and Prevention, Guangxi Key Laboratory of Reproductive Health and Birth Defects Prevention, Maternal and Child Health Hospital, Nanning, China; ^2^ Department of Genetic and Metabolic Central Laboratory, Maternal and Child Health Hospital, Nanning, China; ^3^ Guangxi Clinical Research Center for Birth Defects, Maternal and Child Health Hospital, Nanning, China; ^4^ Neonatal Surgery, Maternal and Child Health Hospital, Nanning, China; ^5^ Guangxi Clinical Research Center for Pediatric Diseases, Maternal and Child Health Hospital, Nanning, China

**Keywords:** intellectual developmental disorder type 61, *MED13* gene, novel variant, whole-exome sequencing, unreported complication

## Abstract

Intellectual developmental disorder type 61 (MRD61) is an extremely rare autosomal dominant disorder caused by variants in the *MED13* gene. This gene encodes a subunit of the mediator complex, which is also known as TRAP, SMCC, DRIP or ARC. This complex functions as a transcriptional coactivator and is essential for the expression of almost all genes. To date, only 26 cases of MRD61 have been reported worldwide. The main symptoms are intellectual disability (ID) of varying degrees, developmental delay (DD), hypotonia during infancy, facial dysmorphism, language impairment, restricted growth, skeletal and limb abnormalities, and behavioral abnormalities. In this study, we recruited a Chinese family in which two individuals were diagnosed with MRD61. Whole-exome sequencing of the proband revealed a novel heterozygous frameshift variant in the *MED13* gene (NM_005121.3): c.5641delinsTC (p.R1882Sfs*9). This variant was inherited from the affected mother and was subsequently confirmed in both the proband and her other family members using Sanger sequencing. The dysmorphology profile of both patients described here is similar to that associated with MRD61. Compared with previously reported cases of MRD61, the proband presented with congenital megacolon, a previously unreported complication. Additionally, skeletal and limb deformities, eye or vision abnormalities, behavioral issues and brain abnormalities were absent in our patients. This is the first report of MED13-associated intellectual developmental disorder-61 in China. This molecular diagnosis expands the known genetic spectrum of MRD61. Furthermore, the specific manifestations observed in these patients with this condition provide valuable additional clinical insight into the syndrome.

## Introduction

Intellectual developmental disorder type 61 (MRD61, MIM 618009) is a very rare autosomal dominant disorder characterized by global developmental delay apparent in infancy, mild to moderate intellectual disability, facial dysmorphism, language impairment, growth restriction, and behavioral abnormalities, including autism spectrum disorder (ASD) and attention-deficit hyperactivity disorder (ADHD) (1). The disease is caused by heterozygous mutations in the *MED13* gene (NM_005121.3; OMIM 603808), which is located on chromosome 17q23.2. This 30-exon gene encodes a subunit of the mediator complex, which is also referred to as TRAP, SMCC, DRIP, or ARC (2). The Mediator complex, a large multiprotein complex universally expressed in eukaryotes, plays a crucial role in transcription regulation by stabilizing the preinitiation complex and communicating transcription-activating signals to RNA Polymerase II, thus promoting the transcription of all protein-coding and most nonprotein-coding genes (3–5). A key component of this complex is the four-subunit kinase module, composed of MED13, MED12, and cyclin-dependent kinase 8 (CDK8), along with their respective paralogues MED13l, MED12l, and CDK19, which function together with Cyclin C (3, 4). This kinase module, also known as the “Module”, is involved in both transcriptional activation and repression. It has been reported to prevent the association of the Mediator with the RNA Polymerase II preinitiation complex, thereby inhibiting transcription initiation and/or re-initiation (6). Additionally, the Module may activate transcription (7). MED13, in particular, facilitates the reversible physical interaction between the Module and the Mediator in both humans and yeast (4, 8, 9). Given its critical role in transcription, *MED13* is a compelling candidate gene for neurodevelopmental disabilities, which are often associated with variants affecting the Module genes and are broadly termed “transcriptomopathies” (10, 11).

To date, only 26 patients carrying *MED13* variants have been documented in the literature (1, 12–20). The mechanisms by which distinct *MED13* mutations produce divergent clinical phenotypes remain unknown. Additional case reports documenting MED13 mutations and their phenotypic manifestations will clarify both the disease pathology and its genotype-phenotype correlations. In this study, we identified a novel frameshift mutation in the *MED13* gene in a Chinese family with MRD61. The patient inherited this variant from her affected mother. The clinical phenotypes observed in both the patient and her mother were thoroughly characterized. This report expands the spectrum of *MED13* gene variants and provides additional molecular and clinical information, which helps to further enhance the understanding of MRD61 syndrome.

## Materials and methods

### Patients

**Figure 1:**
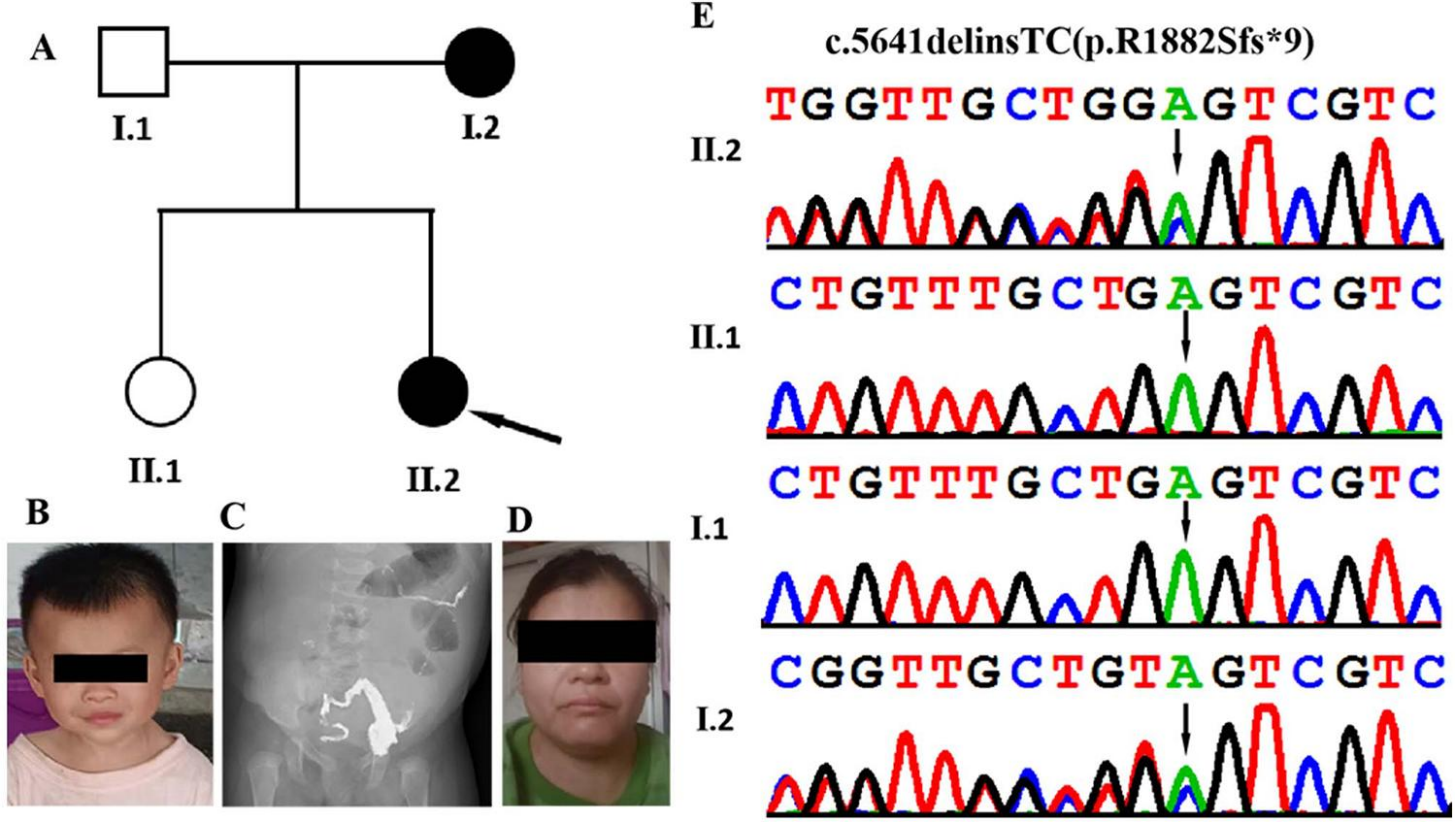
Clinical and genetic features. **(A)** Family pedigree showing that both the proband and her mother and affected. **(B)** Facial appearance of the proband (II-2) at the age of 2 years and 3 months, showing hypertelorism, everted lower eyelids, depressed nasal bridge, broad nasal tipand, and broad philtrum. **(C)** Abdominal x-ray acquired at 2 months and 23 days in the proband (II-2) show congenital megacolon. **(D)** Facial appearance of the proband's motherexhibiting epicanthal folds, a long philtrum, and a wide mouth. **(E)** DNA sequence chromatograms from Sanger sequencing of *MED13*, showing showing a heterozygous frameshift variant *MED13* (NM_005121.3): c.5641delinsTC (p.R1882Sfs*9) in the proband. Sanger sequencing further revealed that his affected mother was heterozygous for the same variant and that his father and older sister were normal.

A Chinese family with aganglionic megacolon and developmental delay was referred to the Paediatric Surgery Department of Guangxi Maternal and Child Health Hospital for genetic evaluation (Figure 1A). The Institutional Review Board and Ethics Committee of Guangxi Maternal and Child Health Hospital approved the study protocol, which adhered to the principles of the Declaration of Helsinki. The parents of the affected individuals provided written informed consent for publishing clinical data and photographs.

### Whole exome sequencing and sanger sequencing

Peripheral blood lymphocytes (2 mL) were collected from the patient and his family members. Genomic DNA was subsequently extracted from these samples with a Lab Aid DNA kit (Zeshan Biotechnology Co., Ltd., Xiamen, China) according to the manufacturer's instructions. Whole-exome sequencing (WES) was achieved using the Agilent SureSelect V5 enrichment capture kit (Agilent Technologies, Santa Clara, CA, USA) and analyzed using an Illumina HiSeq 2000 with 100 bp paired-end sequencing (Illumina, San Diego, California, USA). The sequencing reads were aligned to the human reference genome (hg19/GRCh37) using the Genome Analysis Toolkit (GATK, version 3.4; Broad Institute, Cambridge, MA, USA). The TGex software (http://tgex.genecards.org/) was used for variant calling and annotation. Variants with a minor allele frequency (MAF) of ≤0.001 in public databases (the 1000 Genomes Project, the Exome Sequencing Project and the Exome Aggregation Consortium) were prioritized. In silico tools (REVEL, PolyPhen2, SIFT, LRT, Mutation Assessor, CADD, and MutationTaster) were used to predict the functional impact of candidate variants. The pathogenicity of these variants was evaluated according to the American College of Medical Genetics and Genomics (ACMG) guidelines and the ClinGen Sequence Variant Interpretation (SVI) Working Group recommendations (21).

## Result

The proband was a Chinese girl who was the second child of non-consanguineous parents (Figures 1A,B). The patient was born vaginally at 39 weeks gestation following an uncomplicated spontaneous labor. Birth measurements included a weight of 3.3 kg (75th percentile), length of 50 cm (80th percentile), and head circumference of 34 cm (50th percentile). APGAR scores were 9 at 1 min, and 10 at both 5 and 10 min. At two months of age, the infant was admitted to a local hospital with recurrent fever and diarrhea. After showing no improvement following one week of initial treatment, the patient was transferred to our hospital for further evaluation and management. She was subsequently diagnosed with congenital megacolon and enteritis (Figure 1C). She underwent radical surgery for the megacolon and reversal of the intestinal ostomy. Subsequently, she was admitted to hospital several times due to intestinal obstruction and constipation. During her first year, she exhibited feeding difficulties, failure to thrive and hypotonia. Her developmental milestones were also slightly delayed. She achieved head control at 5 months, started crawling at 9 months, and began walking with support at 17 months. Her language development has been delayed; she did not speak her first word until she was around 20 months old, and she is currently unable to speak in complete sentences. At 10 months of age, the Gesell Developmental Diagnostic Scale measured her Developmental Quotient (DQ; scores <70 indicate delay), yielding the following domain scores: gross motor (70), fine motor (68), adaptive (65), language (48), and personal-social (67). Her most recent examination, at the age of 2 years and 3 months, revealed postnatal growth retardation, short stature (height: 80 cm, <−2 SD) and dysmorphic facial features, which included a hypertelorism, everted lower eyelids, depressed nasal bridge, broad nasal tip, and broad philtrum (Figure 1B).

The patient's family history reveals that her mother experienced delayed motor and language development during childhood. Specifically, she began walking at 17 months and speaking simple words at 20 months. Currently, at the age of 36, the patient's mother exhibits short stature, with a height of 154 cm (<−2.5 SD). Additionally, she has a borderline intellectual disability (IQ: 78–83), learning difficulties and presents with mild facial dysmorphism, including epicanthal folds, a long philtrum, and a wide mouth (Figure 1D).

To identify the genetic basis of the disease, we conducted whole-exome sequencing on the proband. The sequencing yielded 6.9 Gb of data, covering 98.8% of the target region with 98.5% of targets exceeding 20X read depth. We detected 29,046 single nucleotide variants (SNVs) and insertion/deletion (indel) variants in coding regions and splice sites (within 10 bp of exon boundaries). After excluding synonymous variants and those with a minor allele frequency (MAF) >1% in public databases (1000 Genomes Project, gnomAD, ESP, ExAC) and our internal datasets, 610 variants remained. TGex analysis (LifeMap Sciences, USA) prioritized 9 candidate variants across 7 genes (*MED13*, *POGZ*, *SETBP1*, *SCN9A*, SCO1, *APTX*, *TIE1*) associated with phenotypes including congenital megacolon, intellectual disability, motor delay, and developmental delays. Heterozygous variants in *SCO1* and *APTX* were excluded as their associated disorders follow autosomal recessive inheritance. Variants in *POGZ*, *SETBP1*, *SCN9A*, and *TIE1* were paternally inherited from the unaffected father. The analysis revealed a heterozygous frameshift variant in *MED13* exon 25 (c.5641delinsTC, p.R1882Sfs*9) in the proband, which Sanger sequencing confirmed as maternally inherited (Figure 1E). This variant is a novel variant that has not been found in major public databases, including the Exome Sequencing Project, gnomAD database, and 1000 Genomes Project, as well as disease-specific databases such as ClinVar and the Human Gene Mutation Database. This variant (c.5641delinsTC; p.R1882Sfs*9) was located in the exon 25 of the *MED13* gene. Its functional impact likely resembles other reported *MED13* loss-of-function variants, such as the known c.4198C>T(p.Arg400*) mutation. This alteration leads to complete protein loss accompanied by markedly reduced mRNA levels through nonsense-mediated decay. According to the ACMG/AMP standards and guidelines, the variant is classified as ‘likely pathogenic’ based on the PVS1 and PM2 supporting criteria (Table 1). XHMM software and depth-of-coverage analysis detected no pathogenic or likely pathogenic copy number variants in the patient's WES data.

**Table 1:** Predicted pathogenicity of novel MED13 variant.

Gene	Variant (NM_005121.3)	Inheritance	AutoPVS1	Mutationtaster	RDDC	ENTPRISE	ACMG/AMP
MED13	c.5641delinsTC (p.R1882Sfs*9)	Maternal	Very Strong	D	LP	D	LP (PVS1 + PM2_Supporting)

D, deleterious or damaging; LP, Likely pathogenic.

## Discussion

Intellectual developmental disorder type 61 (MRD61) is a very rare autosomal dominant disorder (1). The *MED13* variant was initially detected through whole exome sequencing (WES) in a large group of patients with autism, for whom a detailed clinical description was unavailable (12, 13). Subsequently, Snijders Blok et al. provided a detailed description of the clinical phenotype and molecular genetic features of 13 patients with MED13-related disease (1). To date, only 26 individuals with MRD61 have been reported (Table 2). The clinical manifestations of these patients include facial dysmorphism, global developmental delay in infancy, varying degrees of intellectual disability, language developmental delay, growth restriction, short stature, skeletal abnormalities, and behavioral abnormalities, including autism spectrum disorder and attention-deficit/hyperactivity disorder (1, 12–20). In this study, we conducted WES and detected a heterozygous frameshift variant in the *MED13* gene in a Chinese patient diagnosed with MRD61.

**Table 2:** Clinical characteristic of patients with MED13 novel variants.

Patients clinical data	Samocha et al. 2014	Yuen et al. 2017	Snijders Blok et al. 2018	Kahrizi et al. 2019	De Nardi et al.2021	Rogers et al. 2021	Tolmacheva et al. 2022	Trivisano et al. 2022	Rivera et al. 2024	Al-Sarraj et al. 2024	Yahia et al. 2024	Our patient		Total
Patients	*n* = 2	*n* = 3	*n* = 13	*n* = 1	*n* = 1	*n* = 1	*n* = 1	*n* = 1	*n* = 1	*n* = 1	*n* = 1	*n* = 2		*N* = 28
												Proband	Affected mother	
Variant (NM_005121)	NA	p.Arg512*, p.Ala418Thr, p.Tyr1649*	see (1)	p.Ala443GlufsTer6	p.Pro327Ser)	p.Gly1150Glu	p.Pro835Ser	p. Tyr834Cys	c. 1009 + 8A > G	p.V346I	p.Arg1409Ter			
Gender	1M/1F	2M/1F	4M/9F	F	F	M	M	M	F	NA	NA	F	F	
Age	NA	NA	3 to 32y (median 10y)	8 Y	13 Y	7 Y	2 M#	24 M#	18Y	NA	NA	2Y3M^#^	33Y	
ID and/or DD	2ID	1NA,1DD,1ID	10ID (2 borderline,7mild, 1 Moderate),3DD,	Moderate ID	Mild ID	Severe ID	NA	Severe ID	Severe ID	NA	NA	Mild ID and DD	Borderline ID	23/23
Brain MRI Abnormalities	NA	NA	3/9.	NA	-	-	+	+	-	NA	NA	-	-	5/16.
Delayed motor development	NA	1,2NA	7/13.	NA	+	+	NA	+	+	NA	NA	+	+	14/20
Speech delay or disorder	1,1NA	1,2NA	13/13	NA	+	+	NA	+	+	NA	+	+	+	22/22.
Hypotonia	NA	NA	3/13.	NA	+	+	+	+	+	NA	NA	+	NA	9/19.
ASD and/or ADHD	2	3	8/13.	NA	-	-	NA	NA	+	+	NA	-	-	15/24
Dysmorphic facial features	NA	NA	8/13.	+	+	+	+	+	+	NA	NA	+	+	16/21.
Hearing loss	NA	NA	2/13.	NA	+	-	NA	+	-	NA	NA	-	-	4/19.
Eye/vision abnormalities	NA	NA	8/13.	NA	+	-	+	+	NA	NA	NA	-	-	11/19.
skeletal and limb abnormalities	NA	NA	5/13.	NA	+	+	NA	NA	+	NA	NA	-	-	8/18.
Growth delay/restriction	NA	NA	-	NA	+	+	+	+	NA	NA	NA	+	NA	
Feeding difficulties	NA	NA	NA	NA	+	NA	+	dysphagia	Dysphagia	NA	NA	+	NA	
Chronic obstipation	NA	NA	4/13.	NA	-	NA	NA	NA	+	NA	NA	+	-	6/17.
Congenital heart abnormalities	NA	NA	3/13	NA	-	-	+	NA	NA	NA	NA	-	-	
Other	NA	Chronic sleep disturbance	2 Microcephaly,2 short stature, 2 Chronic sleep disturbances,1precocious puberty,1 seizures	Microcephaly	IgA deficit	Macrocephaly,short stature	Ileal atresia, bronchopulmonary dysplasia, neonatal death	Congenital hypotiroidism,Microcephaly,seizures	Respiratory problems, food allergies to lactose and colic, inverted nipples and clitoromegaly	NA	NA	Congenital megacolon,Short stature	Short stature	

To explore the genetic and clinical characteristics of MRD61, we conducted a comprehensive review of literature on the clinical manifestations and genetic characteristics of patients with *MED13* variants (Table 2). Statistical analysis revealed that, to date, only 28 affected individuals from 26 unrelated families (including our patients) have been reported to carry *MED13* gene variants worldwide (1, 12–20). These patients exhibit highly heterogeneous clinical manifestations across multiple domains. However, certain common features (>50% of cases) can still be identified. All patients (23/23) have intellectual disability or and developmental delay. Notably, the severity of these conditions is relatively mild, with only five patients showing moderate to severe intellectual impairment. Our patients also present with mild intellectual disability and motor developmental delay. In the language domain, all patients (22/22) exhibit language developmental delay, with about half of them having significant language disorders. This finding indicates that language disorders are a typical feature of MRD61. Most patients (14/20) experience motor delay or impairment. Although some patients do not learn to walk until after 4.5 years of age, all patients eventually acquire the ability to walk. Hypotonia in infancy is also relatively common (9/19). Almost all patients (16/21) show facial dysmorphic features, but there is a lack of consistency—some patients only present with wide-set eyes, narrow eyelids, and a wide mouth, while others exhibit more pronounced facial deformities. In our patients, the proband shows a marked dysmorphic face, while the affected mother has a milder presentation. Fifteen of the 24 patients reported behavioral problems, including autistic behavior and attention deficit hyperactivity disorder (ADHD). Notably, two patients in this study had no behavioral problems. Skeletal and limb abnormalities were observed in eight patients (8/18), including high-arched feet, spinal deformities (scoliosis/kyphosis), hip dysplasia/joint hypermobility, and toe deformities. Another point of concern is eye/vision abnormalities; more than half of the patients have such impairments, and early corrective treatment can improve the prognosis of children. In addition, these patients present a wide variety of symptoms. Among these patients, five exhibited growth restriction, which may be partly attributed to feeding difficulties and gastrointestinal issues. All five of these patients experienced feeding difficulties, with one patient diagnosed with ileal atresia and the proband in this study presenting with a previously unreported case of congenital megacolon. Some patients had brain abnormalities (5/16), chronic constipation (6/17 cases), deafness (4/19 cases), and congenital heart abnormalities (4/18 cases). Other dysmorphic features included IgA deficiency, seizures, congenital hypothyroidism, microcephaly, macrocephaly, short stature, chronic sleep disorders, ileal atresia, bronchopulmonary dysplasia, respiratory problems, lactose intolerance/food allergies, colic, and congenital megacolon. In contrast, the patients in this group did not present with these diverse dysmorphic features, including skeletal and limb deformities, eye/vision abnormalities, behavioral problems, brain abnormalities, and other rare dysmorphic features.

To date, a total of 25 *MED13* variants (including ours) associated with MRD61 have been reported (1, 12–20). These variants include 14 missense and in-frame variants, as well as 12 truncating variants (e.g., nonsense, frameshift, and splicing variants that result in protein frameshifts) (Table 2). The type of genetic variant is often linked to the severity of the associated clinical phenotype. According to the literature, missense variants in the *MED13* homolog gene *MED13l* have been reported to correlate with more severe phenotypes, including a higher incidence of seizures, MRI abnormalities, autism spectrum features, and cardiac abnormalities (22). Consistent with the literature on *MED13l*, we observed that severe intellectual disability was only present in two MRD61 patients with missense mutations and one MRD61 patient with a splicing site mutation (potentially in-frame). However, this observation has not reached statistical significance. Furthermore, the presence of other clinical features, such as delayed language development, skeletal and limb abnormalities, seizures, congenital heart defects, and brain abnormalities, does not appear to be dependent on the type of mutation. Additionally, patients carrying the same genetic mutation may also exhibit a variety of phenotypes. For example, two unrelated patients carrying the same mutation p.Pro327Ser show significant phenotypic differences. Although both patients have intellectual disability, delayed language and motor development, deafness, skeletal and limb abnormalities, and hypotonia, the patient described by De Nardi et al. has prominent facial dysmorphism similar to Kabuki syndrome and more extensive skeletal and limb abnormalities, while the patient reported by Snijders Blok et al. does not have significant facial or skeletal and limb abnormalities but presents with additional cardiac phenotypes, manifested as aortic root and pulmonary artery dilation (1, 16). Moreover, within the same family, the clinical manifestations of different patients are not the same, and it seems that the condition is more severe in the next generation. In the cases reported by Snijders Blok et al. and in this study, the affected mothers have normal or borderline intelligence, with mild facial dysmorphism and skeletal and limb abnormalities; whereas the affected children exhibit mild intellectual disability, feeding difficulties, megacolon, more extensive skeletal and limb abnormalities, and more pronounced facial dysmorphism (1). Therefore, patients with heterozygous mutations in the *MED13* gene exhibit highly diverse clinical manifestations, and no clear correlation between genotype and phenotype has been established so far. The exact mechanisms by which these mutations lead to MRD61 remain unclear. According to Snijders Blok et al., the disease may be caused by multiple mechanisms (1). On the one hand, loss-of-function mutations may lead to haploinsufficiency of the *MED13* gene. On the other hand, missense mutations in *MED13* may disrupt or introduce new phosphorylation sites (e.g., casein kinase 1 phosphorylation sites), thereby affecting the protein-protein interactions of the forkhead-associated domain involved in various important cellular functions. Further case reports and functional studies are crucial for our understanding of the disease and its potential molecular mechanisms.

In summary, we identified a novel frameshift mutation in the *MED13* gene in a Chinese family with MRD61. This is the first report of a Chinese family with the MED13 variant. The proband in this family exhibited a clinical phenotype similar to MRD61, including mild intellectual disability, developmental delay, delayed language development, growth restriction, short stature, facial dysmorphism, feeding difficulties, hypotonia during infancy, chronic constipation, and congenital megacolon. The affected mother only presented with borderline intellectual disability, mild language impairment, mild facial dysmorphism, and short stature. The phenotypic diversity caused by *MED13* variants suggests that in-depth investigation of the specific functions of these variants will help deepen the understanding of the disease and its pathogenesis. Moreover, these data further expand the mutational and phenotypic spectrum of MRD61.

## Data Availability

All data that support the findings of the current study are available from the corresponding author upon reasonable request.

## References

[1] Blok LS Hiatt SM Bowling KM Prokop JW Engel KL Cochran JN *de novo* mutations in MED13, a component of the mediator complex, are associated with a novel neurodevelopmental disorder. Hum Genet. (2018) 137(5):375–88. 10.1007/s00439-018-1887-y 29740699 PMC5973976

[2] Sato S Tomomori-Sato C Parmely TJ Florens L Zybailov B Swanson SK A set of consensus mammalian mediator subunits identified by multidimensional protein identification technology. Mol Cell. (2004) 14(5):685–91. 10.1016/j.molcel.2004.05.006 15175163

[3] Calpena E Hervieu A Kaserer T Swagemakers SMA Goos JAC Popoola O *de novo* missense substitutions in the gene encoding CDK8, a regulator of the mediator complex, cause a syndromic developmental disorder. Am J Hum Genet. (2019) 104(4):709–20. 10.1016/j.ajhg.2019.02.006 30905399 PMC6451695

[4] Harper TM Taatjes DJ. The complex structure and function of mediator. J Biol Chem. (2018) 293(36):13778–85. 10.1074/jbc.R117.794438 28912271 PMC6130968

[5] Soutourina J. Transcription regulation by the mediator complex. Nat Rev Mol Cell Biol. (2018) 19(4):262–74. 10.1038/nrm.2017.115 29209056

[6] Knuesel MT Meyer KD Bernecky C Taatjes DJ. The human CDK8 subcomplex is a molecular switch that controls mediator coactivator function. Genes Dev. (2009) 23(4):439–51. 10.1101/gad.1767009 19240132 PMC2648653

[7] Conaway RC Conaway JW. Function and regulation of the mediator complex. Curr Opin Genet Dev. (2011) 21(2):225–30. 10.1016/j.gde.2011.01.013 21330129 PMC3086004

[8] Fant CB Taatjes DJ. Regulatory functions of the mediator kinases CDK8 and CDK19. Transcription. (2019) 10(2):76–90. 10.1080/21541264.2018.1556915 30585107 PMC6602567

[9] Tsai KL Sato S Tomomori-Sato C Conaway RC Conaway JW Asturias FJ. A conserved mediator-CDK8 kinase module association regulates mediator-RNA polymerase II interaction. Nat Struct Mol Biol. (2013) 20(5):611–9. 10.1038/nsmb.2549 23563140 PMC3648612

[10] Caro-Llopis A Rosello M Orellana C Oltra S Monfort S Mayo S *de novo* mutations in genes of mediator complex causing syndromic intellectual disability: mediatorpathy or transcriptomopathy? Pediatr Res. (2016) 80(6):809–15. 10.1038/pr.2016.162 27500536

[11] Poot M. Mutations in mediator complex genes CDK8, MED12, MED13, and MEDL13 mediate overlapping developmental syndromes. Mol Syndromol. (2020) 10(5):239–42. 10.1159/000502346 32021594 PMC6997798

[12] Yuen RKC Merico D Bookman M Howe JL Thiruvahindrapuram B Patel RV Whole genome sequencing resource identifies 18 new candidate genes for autism spectrum disorder. Nat Neurosci. (2017) 20(4):602–11. 10.1038/nn.4524 28263302 PMC5501701

[13] Samocha KE Robinson EB Sanders SJ Stevens C Sabo A McGrath LM A framework for the interpretation of *de novo* mutation in human disease. Nat Genet. (2014) 46(9):944–50. 10.1038/ng.3050 25086666 PMC4222185

[14] Trivisano M De Dominicis A Micalizzi A Ferretti A Dentici ML Terracciano A MED13 Mutation: a novel cause of developmental and epileptic encephalopathy with infantile spasms. Seizure. (2022) 101:211–7. 10.1016/j.seizure.2022.09.002 36087421

[15] Rogers AP Friend K Rawlings L Barnett CP. A *de novo* missense variant in MED13 in a patient with global developmental delay, marked facial dysmorphism, macroglossia, short stature, and macrocephaly. Am J Med Genet A. (2021) 185(8):2586–92. 10.1002/ajmg.a.62238 33931951

[16] De Nardi L Faletra F D'Adamo AP Bianco AMR Athanasakis E Bruno I Could the MED13 mutations manifest as a kabuki-like syndrome? Am J Med Genet A. (2021) 185(2):584–90. 10.1002/ajmg.a.61994 33258286

[17] Tolmacheva E Bolshakova AS Shubina J Rogacheva MS Ekimov AN Podurovskaya JL Expanding phenotype of MED13-associated syndrome presenting novel *de novo* missense variant in a patient with multiple congenital anomalies. BMC Med Genomics. (2024) 17(1):130. 10.1186/s12920-024-01857-z 38745205 PMC11094910

[18] Rivera MD Aponte SN Rivera F Arciniegas NJ Carlo S. MED13 gene mutation related to autism Spectrum disorder: a case report. Cureus. (2024) 16(5):e59904. 10.7759/cureus.59904 38854223 PMC11157474

[19] Al-Sarraj Y Taha RZ Al-Dous E Ahram D Abbasi S Abuazab E The genetic landscape of autism spectrum disorder in the middle eastern population. Front Genet. (2024) 15:1363849. 10.3389/fgene.2024.1363849 38572415 PMC10987745

[20] Yahia A Li D Lejerkrans S Rajagopalan S Kalnak N Tammimies K. Whole exome sequencing and polygenic assessment of a Swedish cohort with severe developmental language disorder. Hum Genet. (2024) 143(2):169–83. 10.1007/s00439-023-02636-z 38300321 PMC10881898

[21] Richards S Aziz N Bale S Bick D Das S Gastier-Foster J ACMG laboratory quality assurance committee. Standards and guidelines for the interpretation of sequence variants: a joint consensus recommendation of the American college of medical genetics and genomics and the association for molecular pathology. Genet Med. (2015) 17(5):405–24. 10.1038/gim.2015.30 25741868 PMC4544753

[22] Yi Z Zhang Y Song Z Pan H Yang C Li F Report of a *de novo* c.2605C>T (p.Pro869Ser) change in the MED13l gene and review of the literature for MED13l-related intellectual disability. Ital J Pediatr. (2020) 46(1):95. 10.1186/s13052-020-00847-y 32646507 PMC7350599

